# Bio-based unsaturated polyester resin from post-consumer PET†

**DOI:** 10.1039/d3ra08500g

**Published:** 2024-03-13

**Authors:** David Rubeš, Jaromír Vinklárek, Štěpán Podzimek, Jan Honzíček

**Affiliations:** a Institute of Chemistry and Technology of Macromolecular Materials, Faculty of Chemical Technology, University of Pardubice Studentská 573 532 10 Pardubice Czech Republic jan.honzicek@upce.cz; b Department of General and Inorganic Chemistry, Faculty of Chemical Technology, University of Pardubice Studentská 573 532 10 Pardubice Czech Republic

## Abstract

This study explores the utilization of post-consumer poly(ethylene terephthalate) (PET) as a material in the synthesis of styrene-free unsaturated polyester (UP) resin. The process involves glycolysis of PET waste with diethylene glycol and condensation polymerization with bio-based itaconic acid. The resulting unsaturated polyester possesses reactive methylidene functions that, in contrast to commonly employed fumarates/maleates, facilitate copolymerization with non-styrene reactive diluents. To formulate the resins, methacrylates and itaconates were used for dilution, and the curing process is achieved through a redox initiation system at room temperature, followed by post-curing at elevated temperatures. The cured formulations were characterized by their glass transition temperature, determined by DMA analysis. Mechanical properties were evaluated using standardized tests in tension, flexure, and compression. Particularly promising characteristics are observed in formulations incorporating bio-based dimethyl itaconate (DMI), allowing the formulation of materials with a high ultimate flexural strength (*σ*_f,max_ = 161.4 MPa) and compressive yield point (*σ*_c,yield_ = 131.3 MPa). Furthermore, the low volatility of DMI addresses the health, safety, and ecological concerns associated with the commonly used styrene. This technology not only presents a promising avenue for sustainable UP resin for glass fiber reinforced composites but also allows upcycling PET waste.

## Abstract 2

Styrene-free unsaturated polyester resin synthesized from post-consumer PET is described.

## Graphical abstract

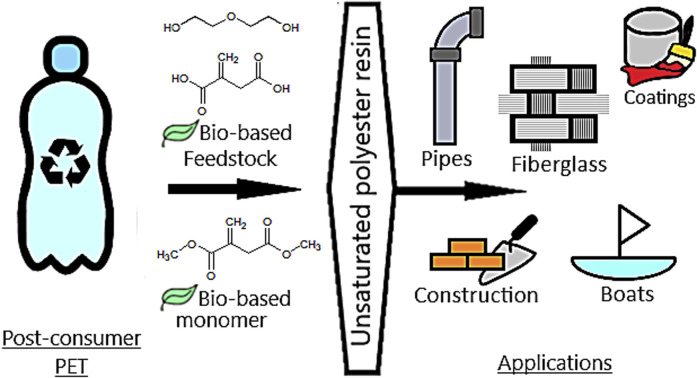

## Introduction

Recycling plastic waste has become one of the promising solutions to environmental concerns related to the mass production of disposable single-use items, as they offer valuable feedstock for industrial production.^1^ However, existing recycling methods, based mainly on remolding and reshaping (mechanical recycling), are predominantly applied to high-density polyethylene (HDPE) and poly(ethylene terephthalate) (PET) and have some limitations.^2,3^ The properties of polymeric materials undergo degradation through multiple reprocessing cycles, resulting in a reduction in molar mass. This effect persists even when using pure and well-sorted polymer blends.^2,4^ Consequently, these factors limit the application of recycled materials to lower value uses (downcycling), deviating from the principles of a circular economy.^5^

The limitations associated with mechanical recycling can be addressed through chemical and biochemical processes that recover monomers, known as chemical recycling.^6–9^ This approach is advantageous for thermoplastic polyesters (*e.g.*, PET and polylactic acid) mainly due to their lower thermodynamic stability compared to polyolefins.^2,6^ The availability of terephthalic acid and its derivatives originating from post-consumer PET serves as a promotor for the development of sustainable thermoplastic^10^ and thermosetting polymers.^11–13^ Furthermore, their production can use partially depolymerized PET that contains oligomeric byproducts, thus reducing secondary waste generation and lowering energy requirements.^14^ In principle, the use of post-consumer PET in this way aligns with the concept of upcycling, as the resulting thermosets represent compounds of enhanced value.^15–17^

Our interest in sustainable UP resins for fiber reinforcement, aimed at reducing greenhouse gas emissions, aligns with prior research in the field. Many studies have explored the use of bio-based itaconic acid (IA)^18–22^ bio-based muconic acid,^23–25^ or bio-derivable fumaric acid^25^ as sources of unsaturation in the polyester backbone, as opposed to oil-based maleic anhydride (MA). The hard building blocks in traditional UPs, derived from phthalate moieties, can be easily replaced by bio-based 2,6-furanedicaroboxylic acid (FDCA)^19,26,27^ or glycolyzed PET.^25^

Reactive diluent (RD) plays a crucial role in the design of UP resins, as it typically represents 25–45% of their mass.^28^ The dominant use of styrene (STY) is driven by its low cost, high dissolution capacity, and favorable reactivity ratios.^29^ However, the urgent need for its replacement arises due to health,^30^ environmental^31^ and safety concerns.^32^ Various alternative RDs have been explored, including acrylates,^19,33^ methacrylates,^19,26,27,29^ itaconates,^20–22,34^ bio-based allylbenzene congeners^35^ and bio-based styrene congeners.^36^ Despite the introduction of several promising concepts for sustainable UP resins in recent years,^21,25–27,33^ none has been successfully commercialized, likely due to high prices and/or limited availability of key components (*e.g.*, muconic acid, FDCA, bio-based RDs).

The aim of this study is to design sustainable UP resins using readily available raw materials and to describe their mechanical properties. To achieve this goal, bio-based IA was selected as a source of unsaturation of polyester, given its recent availability on an industrial scale^37^ and the accompanying price reduction.^38^ RDs based on low-molecular weight methacrylates and itaconates are chosen due to their sufficient reactivity and close-to-unity reactivity ratios. The hard building blocks required for achieving high-performance products with a high glass transition temperature (*T*_g_) are introduced through partially glycolyzed post-consumer PET.

## Results and discussion

### Glycolysis of post-consumer PET

Colorless PET, sourced from post-consumer bottles, was glycolyzed using diethylene glycol (DEG). This choice of diol, known for its high boiling point (245 °C), facilitates the depolymerization process. Furthermore, DEG introduces flexible aliphatic segments into the resulting product, which will be useful for tuning the mechanical properties of the final resin.

**Fig. 1:**
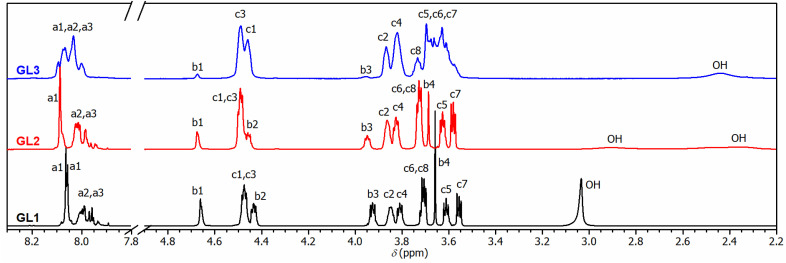
^1^H-NMR spectra of glycolyzed PET under different conditions with assignment of resonances to building blocks given in Scheme 1. In GL3, signals denoted C3, C4, C5, C6, and C7 cover segments of higher EG oligomers.

**Scheme 1:**
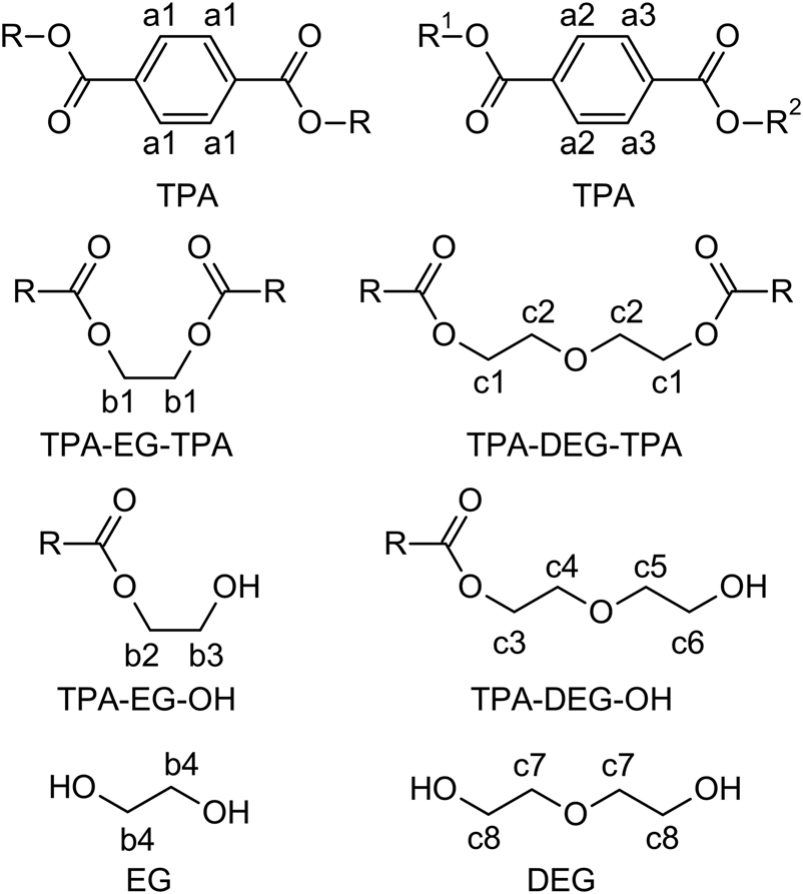
Building blocks present in glycolysates GL1–GL3 and numbering of hydrogen atoms.

**Fig. 2:**
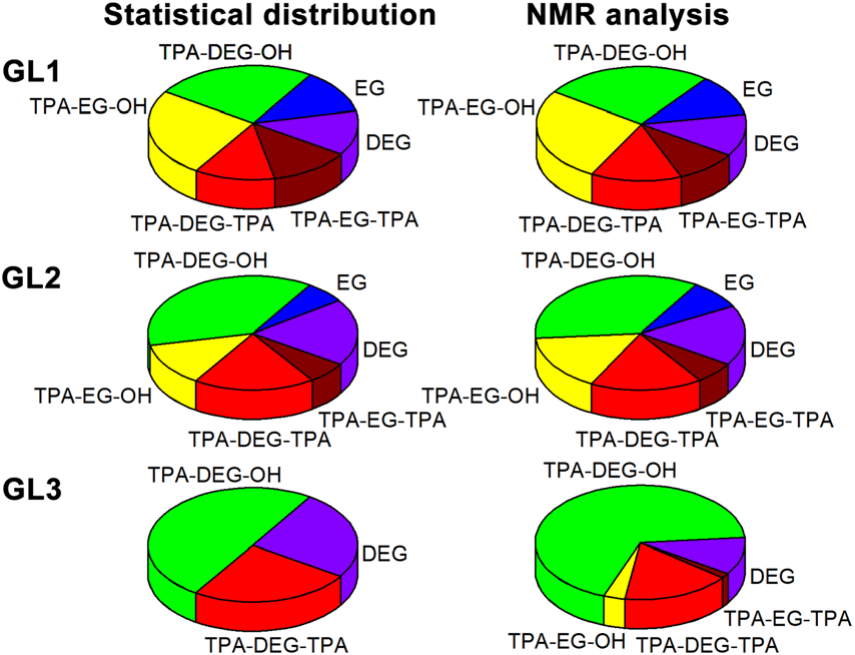
Distribution of the EG and DEG segments in glycolysates. Expected statistical distribution and composition experimentally determined by ^1^H NMR spectroscopy. Moieties labeled according to Scheme 1. For details, see Table S1 in the ESI.†

In the initial experiment, PET was glycolyzed with one molar equivalent of DEG (relative to the repeat unit of PET, *M*_r_ = 192.17 g mol^−1^), resulting in a glycolysis product containing terephthalic acid (TPA), ethylene glycol (EG), and DEG segments in a 1/1/1 molar ratio. The semi-solid product GL1, obtained after heating for 4.5 h at 245 °C, contains a mixture of oligomers, as evidenced by SEC chromatography (Fig. S1 in the ESI†). The ^1^H NMR spectrum (Fig. 1) revealed the presence of fully esterified glycols (TPA-EG-TPA, TPA-DEG-TPA), terminal alcohol functions (TPA-EG-OH and TPA-DEG-OH) and free glycols (EG, DEG); see Scheme 1. They are formed in a molar ratio close to a statistical distribution (1/1/2/2/1/1) of free and esterified hydroxy functions (Fig. 2). It should be noted that the spectrum assignment was consistent with data from the literature^39^ and the connectivity of methylene groups in the EG and DEG segments was confirmed by the ^1^H–^1^H COSY experiment.

To achieve products with a higher DEG content while maintaining the same TPA/(EG + DEG) ratio of 1/2, an excess of DEG was used for PET glycolysis and the EG side product was continuously distilled off during the reaction. This procedure led to the glycolysates GL2 and GL3 with TPA/EG/DEG feed compositions of 1/0.5/1.5 and 1/0/2, respectively. The composition of GL2 and GL3 was investigated by ^1^H NMR spectroscopy (Fig. 1), which confirmed that the distribution of the segments in GL2 closely matched the expected statistical distribution. However, GL3 exhibited a significantly higher content of TPA-DEG-OH (Fig. 2), attributed to the stabilization of C_6_H_4_{COO(CH_2_CH_2_O)_2_OH}_2_ by weak interactions. Furthermore, the broadened bands at 3.5–3.7 ppm suggested DEG oligomerization in the final step when residual EG was distilled from the reaction mixture. This interpretation is supported by SEC chromatography (Fig. S2 and S3 in the ESI†). It revealed the oligomeric character of GL2, while GL3 has considerably higher molar weight (*M*_n_ = 1350 g mol^−1^, *Đ* = 1.7).

### Synthesis of UPs from glycolysates

**Fig. 3:**
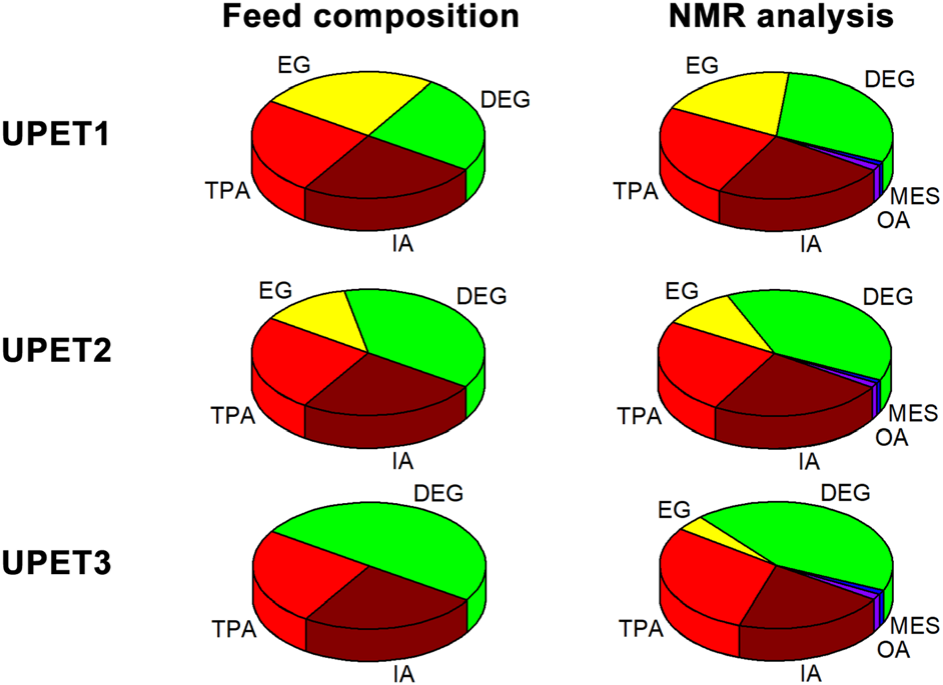
Feed composition of unsaturated polyesters (calculated from the starting PET, DEG, and IA) and composition determined by ^1^H NMR spectroscopy. For details, see Table S2 in the ESI.†

The unsaturated polyesters UPET1–UPET3 were synthesized by condensation polymerization of itaconic acid (IA) with glycolysates GL1–GL3, respectively. These glycolysates introduced both rigid aromatic moieties from PET and flexible aliphatic segments from DEG into the polyester chain. The overall flexibility of the polymeric chains was tuned by variation of the EG/DEG ratio (Fig. 3). Condensation polymerization was carried out in a toluene solution at ∼160 °C, catalyzed by [Bu_2_SnO] to an acid value (AV) of ∼50 mg of KOH/g (Table 1).

**Table 1:** Properties of synthesized UPs

Polyester	AV (mg KOH per g)	*M*_n_ a (g mol^−1^)	*Đ* b
UPET1	44	2150	2.21
UPET2	45	2130	2.03
UPET3	47	2810	2.42

a Number-average molar mass determined by SEC.

b Dispersity determined by SEC. For SEC chromatograms, see Fig. S4–S6 in the ESI.

**Fig. 4:**
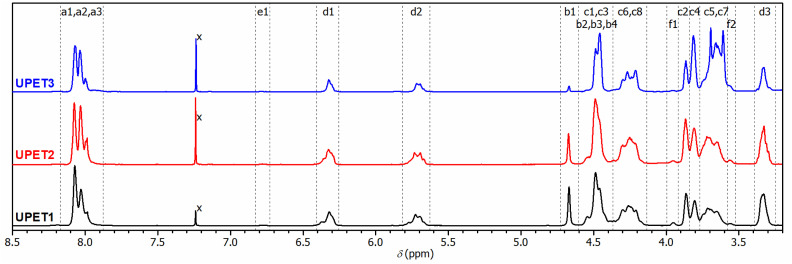
^1^H-NMR spectra of UPs prepared from different glycolysis products with assignment of resonances to given polymer segments shown in Scheme 2. In UPET3, signals denoted C3, C4, C5 and C7 cover segments of higher oligomers of EG.

**Scheme 2:**
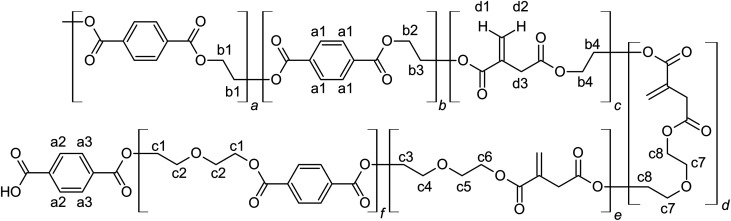
Structure of UPET1–UPET3 polyesters and numbering of hydrogen atoms.

The ^1^H NMR spectra of the resulting UPET1–UPET3 (Fig. 4) confirmed the presence of key building blocks (TPA, IA, EG, and DEG) given in Scheme 2. The assignments were made using the ^1^H–^1^H COSY technique.

**Scheme 3:**
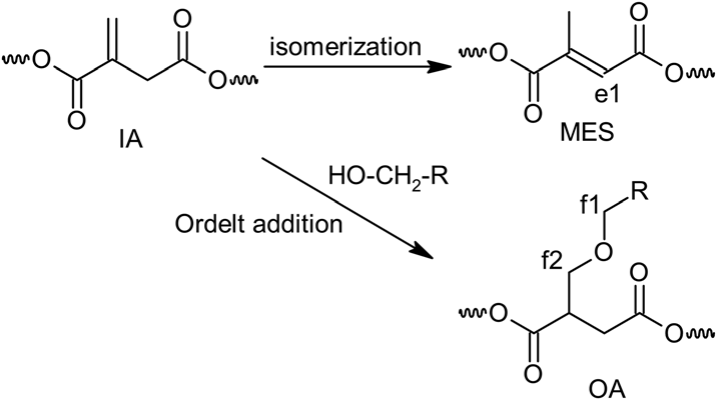
Side reaction upon condensation polymerization with numbering of characteristic hydrogen atoms.

Quantitative analysis of the ^1^H NMR spectra revealed some variations in the content of the building blocks compared to the feed composition, probably due to the loss of EG during the removal of the formed water. The lower content of DEG segments in UPET3 is attributed to the formation of higher EG oligomers during glycolysis. Importantly, ^1^H NMR measurements indicated negligible isomerization of itaconate to mesaconate under the given conditions (Scheme 3 and Fig. 3). The characteristic signal of mesaconate at ∼6.8 ppm is well separated from other UP signals (Fig. 4).^40^ Furthermore, measurements suggested the appearance of Ordelt adducts (Scheme 3),^40^ identified by characteristic resonances at 3.95 and 3.56 ppm. They were assigned to methylene groups that were adjacent to the formed ether function.

According to SEC chromatography, the number-average molar mass (*M*_n_) of the polyesters UPET1 and UPET2 varies around 2150 g mol^−1^ (Table 1), which implies that the UP contains ∼4.5 reactive C=C double bonds per average polyester molecule. The higher value observed for UPET3 (*M*_n_ = 2810 g mol^−1^) is attributed to the contribution of higher oligomers of ethylene glycol (EG) formed during glycolysis.

### Properties of UPET-1 in various RDs

The potential of various monomers to act as RDs, capable of replacing commonly used styrene (STY), was investigated in the formulations of polyester UPET1. Methyl methacrylate (MMA), butyl methacrylate (BMA), dimethyl itaconate (DMI), and dibutyl itaconate (DBI) were selected on the basis of appropriate reactivity ratios, with the aim of ensuring the formation of a well-crosslinked polymer network. Note that, unlike STY, none of these monomers is classified as suspected carcinogen.

Although MMA and BMA are not currently sourced from sustainable feedstocks and can cause harmful skin and eye irritation, they offer higher reactivity than their itaconate analogues.^42^ Another advantage of methacrylates is their low ecotoxicity, as evidenced by higher values of LC_50_ toward *Daphnia magna* compared to STY (Table 2). Although experimental data for DMI for *Daphnia magna* are not available, studies in freshwater plants suggest a behavior similar to that of methacrylates. Even for DBI, which has a lower LC_50_ value for *Daphnia magna*, the ecotoxicity does not exceed that of STY (Table 2). The main advantage of DMI and DBI for industrial applications lies in their very low volatility and flammability (Table 2), and both are produced from sustainable feedstocks.

**Table 2:** Selected properties of itaconate-based UP resins

	*μ* a (mPa s)	*P*_sat_ b (Pa)	FP^c^ (°C)	LC_50_^d^ (mg L^−1^)
UPET1-MMA40	275	3700	10	110
UPET1-MMA30BMA10	274	3037	10	31.2
UPET1-DMI40	2330	4.05	–e	45^f^
UPET1-DMI20MMA20	975	2267	10	45^f^
UPET1-DMI20BMA20	962	114	48.5	31.2
UPET1-DMI20DBI20	3070	2.49	136	6.9
UPET1-STY40	182	667	31	4.9
UPET2-DMI40	1500	4.05	–e	45^f^
UPET2-STY40	196	667	31	4.9
UPET3-DMI40	2700	4.05	–e	45^f^
UPET3-STY40	194	667	31	4.9
UPET1/3-DMI40(3 : 1)	2630	4.05	–e	45^f^
UPET1/3-DMI40(1 : 1)	1110	4.05	–e	45^f^
UPET1/3-DMI40(1 : 3)	2160	4.05	–e	45^f^

a Viscosity of fresh UP resins determined by the Höppler's Rheo–viscosimeter.

b Vapor pressure of RD at 20 °C.^41^ For RD mixtures, the values were calculated using Raoult's law 
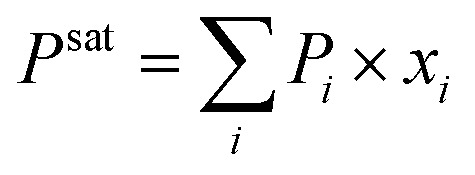
, where *P*_*i*_ and *x*_*i*_ are the vapor pressure of component *i* and its molar fraction in the liquid phase, respectively.

c Flash point of RD.^41^ The value of the most flammable component is given for RD mixtures.

d Ecotoxicity of RD to freshwater algae.^41^ The most toxic component value is given for RD mixtures.

e Nonflammable compound.

f Value for freshwater plants.

Polyester UPET1 exhibits good solubility in methyl esters MMA and DMI. In a formulation with a solid content of 60% by weight, the MMA formulation has a viscosity of 275 mPa s, comparable to the STY solution (182 mPa s). The viscosity of the DMI formulation is higher at 2330 mPa s but remains acceptable for room temperature molding^28^ and 3D printing.^34^ However, butyl esters (BMA and DBI) are only partially miscible with UPET1, attributed to their lower polarity. Consequently, these monomers were used in a mixture with MMA and DMI to fine-tune the mechanical properties. Note that the butyl substituent had only a minor effect on the viscosity of the formulations (Table 2).

**Fig. 5:**
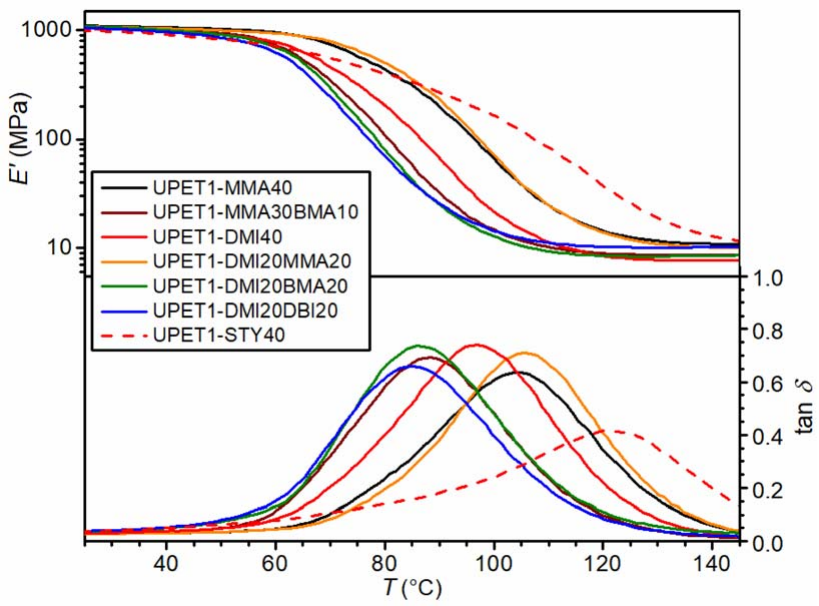
Effect of RD on DMA curves.

Post-cured samples of UPET1 formulations in methacrylates and itaconates exhibit a gel content of more than 95%, indicating the presence of a well-crosslinked polymeric network. This suggestion is supported by values of the crosslink density (*ν*_e_) calculated from the storage modulus in the rubbery region (Table 3). The DMA analysis on this series of samples further revealed a lower glass transition temperature (*T*_g_) compared to the reference formulation diluted with STY of the same solid content, UPET1-STY40 (Fig. 5 and Table 3). This decrease in *T*_g_ is attributed to the absence of “immobile” aromatic moieties in the methacrylate/itaconate segments.

**Table 3:** Mechanical properties of cured itaconate-based UP resins

	*σ*_t,max_ a (MPa)	*E*_t_ b (GPa)	*ε*_t,failure_ c (%)	*σ*_f,max_ d (MPa)	*E*_f_ e (GPa)	*ε*_f,failure_ f (%)	*σ*_c,yield_ g (MPa)	*σ*_c,failure_ h (MPa)	*E*_c_ i (GPa)	*ε*_c,failure_ j (%)	*a*_cU_ k (kJ m^−2^)	Gel^l^ (%)	*T*_g_ m (°C)	*ν*_e_ n (mol dm^−3^)
UPET1-MMA40	83.5 ± 5.0	2.97 ± 0.11	5.1 ± 0.2	122.6 ± 3.4	2.62 ± 0.20	7.3 ± 1.3	111.6 ± 2.1	128 ± 12	2.31 ± 0.14	38.0 ± 2.3	13.2 ± 4.1	99.9	104.3	1.05
UPET1-MMA30BMA10	71.5 ± 5.1	2.97 ± 0.11	3.7 ± 0.7	112.0 ± 7.7	3.16 ± 0.43	7.7 ± 0.7	101.5 ± 3.3	117 ± 12	2.17 ± 0.12	38.2 ± 3.9	11.1 ± 1.4	99.5	88.2	0.84
UPET1-DMI40	85.4 ± 6.9	2.97 ± 0.25	4.6 ± 0.3	140.4 ± 4.5	3.22 ± 0.25	4.9 ± 0.4	120.0 ± 3.1	164 ± 26	2.43 ± 0.14	41.5 ± 4.1	4.5 ± 0.9	98.1	96.9	0.74
UPET1-DMI20MMA20	73.2 ± 4.9	2.86 ± 0.22	3.9 ± 0.4	124.4 ± 2.1	2.85 ± 0.16	8.1 ± 1.5	121.7 ± 2.2	146 ± 16	2.41 ± 0.08	40.3 ± 2.3	8.4 ± 2.9	100.0	105.7	0.96
UPET1-DMI20BMA20	78.9 ± 2.8	2.95 ± 0.11	4.7 ± 0.3	114.6 ± 9.3	2.94 ± 0.61	6.7 ± 0.4	86.6 ± 2.6	131 ± 19	1.76 ± 0.26	39.4 ± 3.9	4.7 ± 0.6	97.9	86.4	0.83
UPET1-DMI20DBI20	59.2 ± 4.7	2.33 ± 0.14	4.2 ± 0.7	96.6 ± 7.0	2.51 ± 0.29	5.3 ± 0.9	71.1 ± 3.0	174 ± 15	1.53 ± 0.11	47.6 ± 1.7	4.8 ± 0.8	96.0	85.0	0.99
UPET1-STY40	82.4 ± 2.1	2.71 ± 0.28	5.3 ± 0.4	115.0 ± 9.5	2.47 ± 0.37	12.1 ± 1.1	105.7 ± 1.9	182 ± 17	2.02 ± 0.14	40.5 ± 2.6	15.6 ± 4.7	96.0	121.3	1.11
UPET2-DMI40	80.7 ± 5.5	2.99 ± 0.19	3.4 ± 0.2	161.4 ± 7.9	3.68 ± 0.34	5.3 ± 0.7	131.3 ± 2.7	171 ± 17	2.46 ± 0.15	37.2 ± 3.3	7.7 ± 1.4	100.0	106.8	0.86
UPET2-STY40	77.2 ± 4.7	2.47 ± 0.22	5.7 ± 0.2	121.9 ± 5.7	2.52 ± 0.37	11.1 ± 2.3	103.9 ± 1.3	154 ± 24	2.08 ± 0.15	37.7 ± 3.9	22.6 ± 5.4	100.0	120.8	1.33
UPET3-DMI40	54.5 ± 0.7	2.18 ± 0.09	6.5 ± 1.1	83.1 ± 2.5	2.12 ± 0.24	17.2 ± 2.7	51.7 ± 1.4	227 ± 36	1.12 ± 0.06	53.1 ± 2.3	12.2 ± 3.6	99.8	84.2	0.74
UPET3-STY40	35.7 ± 1.5	1.22 ± 0.12	14.2 ± 3.0	46.9 ± 2.3	1.10 ± 0.18	> 20 o	75.5 ± 0.7	201 ± 12	1.63 ± 0.05	52.2 ± 1.0	46.0 ± 10.0	93.2	80.4	0.86
UPET1/3-DMI40(3 : 1)	83.4 ± 4.8	3.01 ± 0.16	3.9 ± 0.4	133.7 ± 6.2	3.30 ± 0.18	5.5 ± 0.8	116.6 ± 3.7	185 ± 8	2.28 ± 0.14	41.9 ± 1.7	6.3 ± 1.3	100.0	102.1	0.83
UPET1/3-DMI40(1 : 1)	81.1 ± 2.6	2.95 ± 0.29	4.2 ± 0.4	118.8 ± 3.3	2.99 ± 0.12	7.3 ± 0.7	98.6 ± 2.9	192 ± 7	2.00 ± 0.08	45.0 ± 0.8	8.1 ± 1.7	97.3	96.1	0.84
UPET1/3-DMI40(1 : 3)	65.2 ± 1.9	2.39 ± 0.13	4.5 ± 0.3	86.1 ± 3.2	2.37 ± 0.16	11.5 ± 1.6	75.7 ± 1.5	213 ± 8	1.60 ± 0.08	50.0 ± 0.8	12.4 ± 3.8	96.9	86.6	0.75

a Ultimate tensile strength.

b Tensile modulus.

c Elongation at break.

d Ultimate flexural strength.

e Flexural modulus.

f Flexural strain at break.

g Compressive yield point.

h Compressive stress at break.

i Compressive modulus.

j Compressive strain at break.

k Impact toughness.

l Gel content in cured UP resins.

m Temperature of glass transition determined for UP resins cured by DMA analysis.

n Crosslink density.

o Specimens did not fracture during the experiment.

A *T*_g_ value above 100 °C was observed for the UPET1-MMA40 formulation (104.3 °C). A lower value was observed for UPET1-DMI40 (96.9 °C), attributed to bulky DMI segments, which create a higher free volume in the polymeric network. In the mixed system UPET1-DMI20MMA20, these gaps are effectively filled with more flexible MMA segments, resulting in a considerable increase in the *T*_g_ value (105.7 °C). It is important to note that formulations containing dibutyl esters BMA and DBI give *T*_g_ values below 90 °C due to the internal plasticizing effect of the butyl tails.

### Mechanical properties of UPET1 formulations

Most of the formulations studied showed very similar properties under tension, with an ultimate tensile strength (*σ*_t,max_) of ∼80 MPa, a tensile modulus (*E*_t_) of ∼2.9 GPa, and an elongation at break (*ε*_t,failure_) of ∼4.5%. These values are close to those obtained for the reference formulation UPET1-STY40 (Table 3 and Fig. S7 in the ESI†). A deterioration of mechanical properties was observed only in the case of the UPET1-DMI20DBI20 formulation, where plasticization by DBI led to a decrease in *σ*_t,max_ and *E*_t_, but did not improve *ε*_t,failure_.

**Fig. 6:**
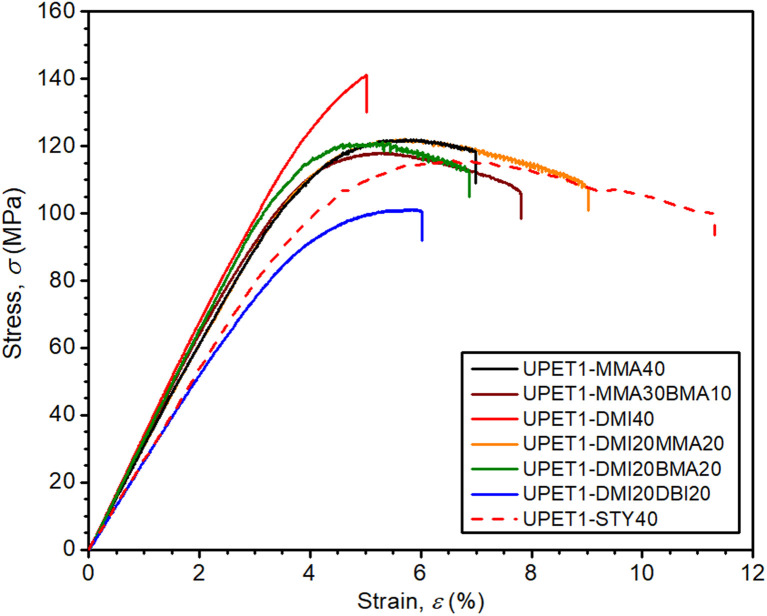
Effect of RD on flexural properties.

The flexural properties of UPET1 appear to be more sensitive to the composition of the RD (Fig. 6 and Table 3). A high ultimate flexural strength was observed for the formulation UPET1-DMI40 (*σ*_f,max_ = 140.4 MPa). However, this improvement, compared to the STY reference, came at the cost of a lower elongation of the outer bending face (*ε*_f,failure_ = 4.9%). Other formulations exhibit ultimate flexural strength (*σ*_f,max_) comparable to UPET1-STY40, but are more brittle, as indicated by lower *ε*_f,failure_ values, ranging between 5.4% and 8.1%. A considerably lower value of *σ*_f,max_ was observed only for UPET1-DMI20DBI20, which correlates with its inferior tensile properties.

**Fig. 7:**
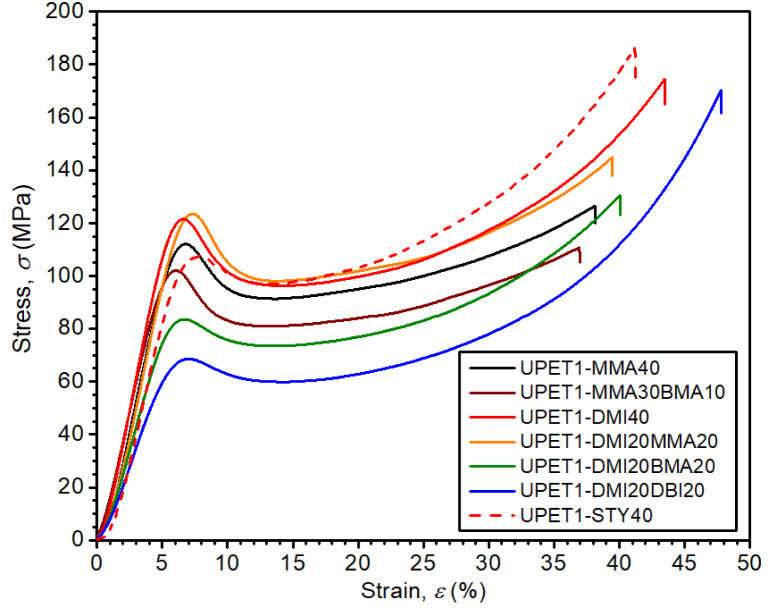
Effect of RD on compressive properties.

The compressive properties of the UPET1 formulations are highly sensitive to the composition of RD (Fig. 7 and Table 3). All formulations show a distinct upper yield point at around 8% strain, but the stress at the yield point (*σ*_c,yield_) varies considerably. The formulations UPET1-DMI40 (120.0 MPa) and UPET1-DMI20MMA20 (121.7 MPa) exhibit higher strength at the yield point than the reference UPET1-STY40 (105.7 MPa) and the formulation UPET1-MMA (111.6 MPa). A significant reduction in this parameter was observed for formulations containing the plasticizing butyl esters BMA or DBI. Interestingly, the stress at break (*σ*_c,failure_) is not directly related to the position of the yield point. High values, comparable to reference UPET1-STY40, were observed for formulations of itaconates UPET1-DMI40 (164 MPa) and UPET1-DMI20DBI20 (174 MPa). It is important to note that the strain at break (*ε*_c,failure_) shows only minor variations between formulations.

Charpy tests revealed high impact toughness (*a*_cU_) for UPET1 formulations in methacrylates. The values obtained for UPET1-MMA40 and UPET1-MMA30BMA10 are comparable to the reference UPET1-STY40 (Table 3). However, formulations of itaconate-based RD exhibit considerably lower impact toughness.

### Properties of cured DMI formulations

To enhance the mechanical properties of the cured UPET1 formulations, modifications to the polyester backbone were used. In this set of experiments, only DMI was selected as RD due to its promising sustainability and safety characteristics, as mentioned above. The STY formulations were again used as references.

**Fig. 8:**
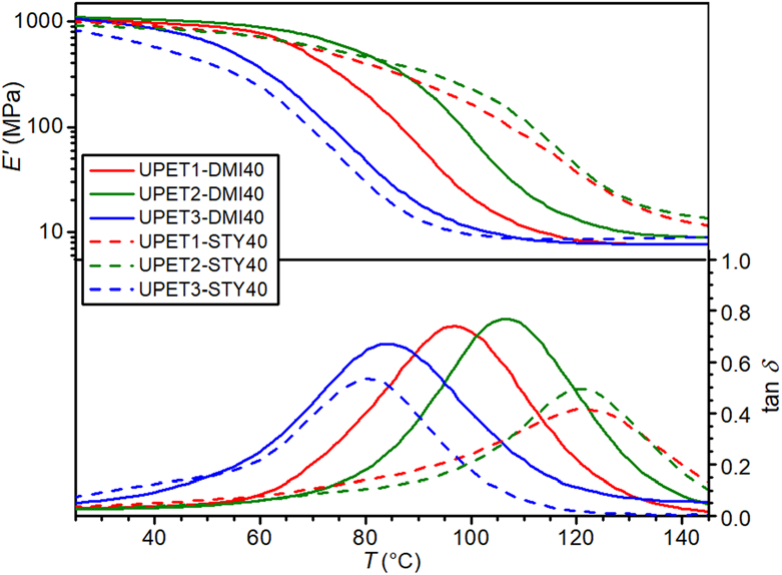
DMA curves of polyesters dissolved in DMI and STY.

In UPET2-DMI40, the partial substitution of the EG segments by DEG unexpectedly led to an increase in the glass transition temperature (*T*_g_) (Fig. 8 and Table 3). This implies a stronger interaction between the polyester chain segments and the RD segments. More flexible DEG segments may fill better the gaps in stiff polyitaconate segments, reducing the free volume in the polymer network. The plasticizing effect of DEG prevails in the UPET3-DMI40 formulations, where the EG segments are fully replaced.

It is interesting that UPET3-STY40 gives a lower *T*_g_ value than UPET3-DMI40, although the opposite trend is reported for polystyrene and poly(dimethyl itaconate) homopolymers.^43,44^ This difference is attributed to the lower polarity of the polyester backbone, which improves the miscibility of the growing polymeric chain with STY. This reduction in phase segregation and polystyrene domain formation contributes to the high *T*_g_ values of UPET1-STY40 and UPET2-STY40. This interpretation is supported by the decrease in the low-temperature shoulder in the damping factor curves (tan *δ*) with increasing DEG content.

**Fig. 9:**
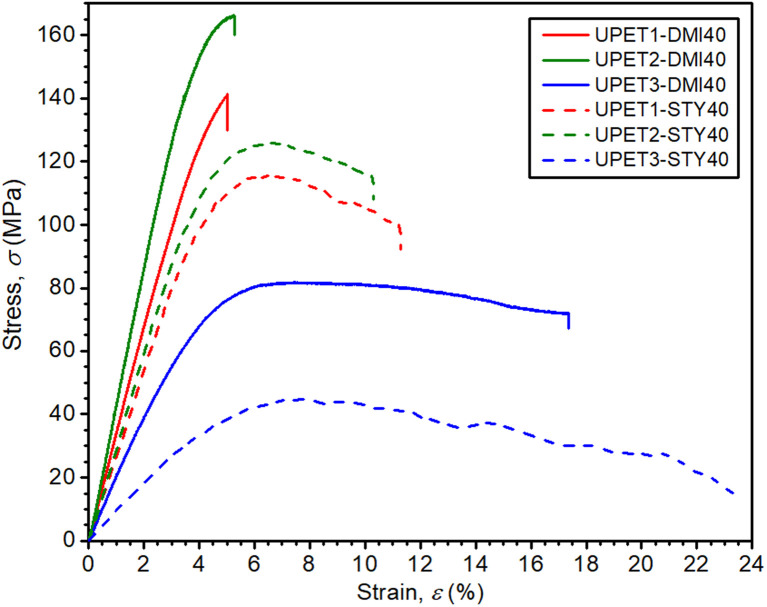
Flexural properties of polyesters dissolved in DMI and STY.

**Fig. 10:**
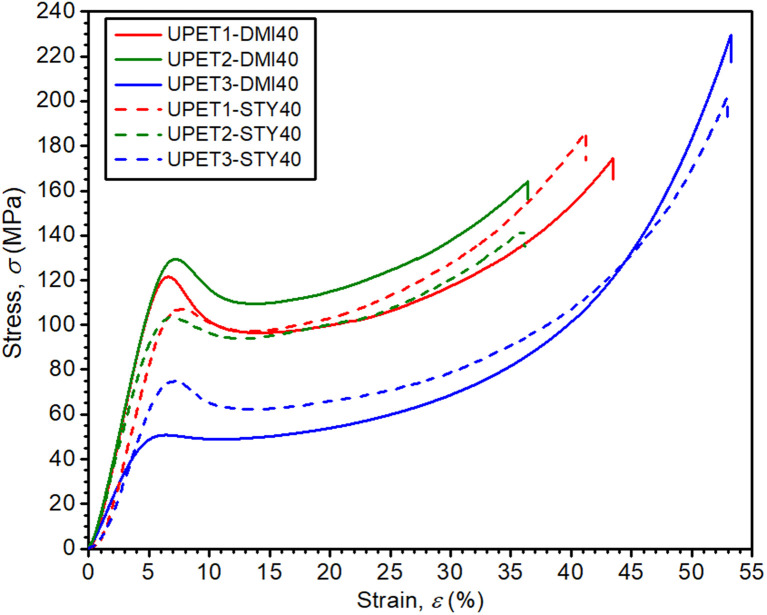
Compressive properties of polyesters dissolved in DMI and STY.

Mechanical tests have revealed that the formulation UPET2-DMI40 exhibits improved bending and compression properties compared to UPET1-DMI40 (Fig. 9 and 10, Table 3). It is demonstrated on a higher ultimate flexural strength (*σ*_f,max_ = 161.4 MPa) and a compressive yield point (*σ*_c,yield_ = 131.3 MPa) without a decrease in flexural deformation at break. Tensile properties remain close to the parent UPET1-DMI40.

In UPET3-DMI40, the plasticizing effect of the DEG segments leads to lower stiffness. The material exhibits considerably higher tensile and flexural strains at break (*ε*_t,failure_ = 6.5%, *ε*_f,failure_ = 17.2%). However, it comes at the cost of lower tensile and flexural strength (*σ*_t,max_, *σ*_f,max_).

In the case of UPET3-STY40, the compressive yield point appears at unexpectedly high stress, compared to UPET3-DMI40. It is probably caused by the considerably lower polarity of the UPET3 prepolymer and corresponds to the unusual thermomechanical behavior mentioned above.

The higher DEG content in the polyester prepolymer significantly improves the impact toughness (*a*_cU_), with a trend that increases in the order UPET1-DMI40 < UPET2-DMI40 < UPET3-DMI40 (Table 3).

It is important to note that plasticization by varying the EG/DEG ratio in the polyester backbone resulted in a material with superior properties compared to those plasticized by mixtures of reactive diluents containing butyl esters (*i.e.*, BMA and DBI).

The inferior properties of these diluents are attributed to the low polarity of the monomers, which reduces their dissolution capacity.

**Fig. 11:**
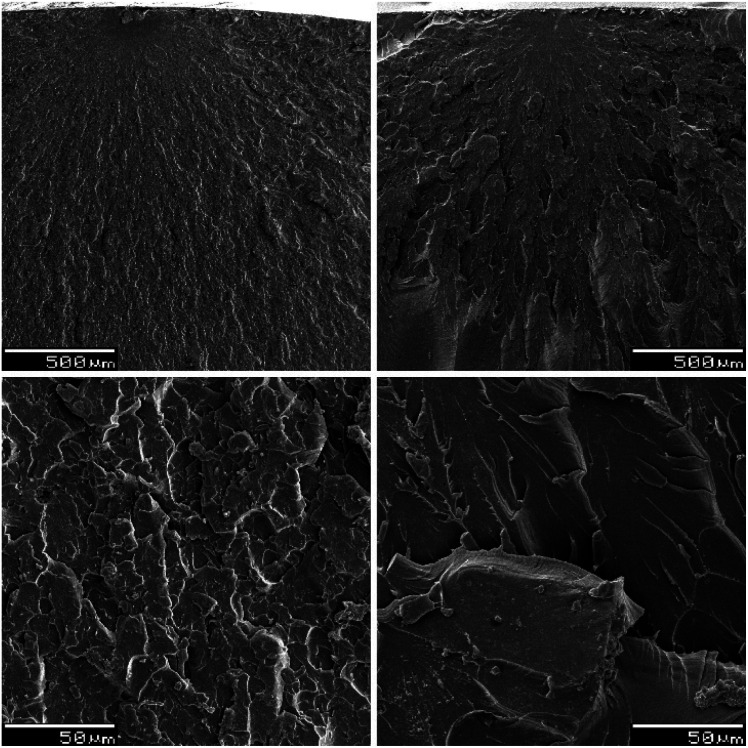
SEM images of UPET2-DMI40 (left images) and UPET2-STY40 (right images). Magnification: 50× (top images) and 500× (bottom images).

Compared to STY reference samples, DMI formulations are generally more brittle. They often exhibit higher ultimate tensile and flexural strength, but lower elongation at break and lower impact toughness. The morphology of the fracture surfaces, obtained during impact tests, was investigated by SEM microscopy on the formulations UPET2-DMI40 and UPET2-STY40. The SEM images in Fig. 11 show the points where the fracture began, along with adjacent mist and hackle zones. The magnified hackle zones (bottom images in Fig. 11) reveal the absence of plastic deformations and fibrils, typical of brittle fractures. In UPET2-DMI40, the impact energy causes the material to disintegrate in the hackle zone, as is evident from the fine structures observed. The more resistant UPET2-STY40 shows macrocracks without signs of crumbling.

Furthermore, it was shown that mechanical properties can be easily tuned by mixing the UPET1-DMI40 and UPET3-DMI40 formulations. The blends, denoted UPET1/3-DMI40, were obtained by mixing the components in weight ratios of 3/1, 1/1, and 1/3. They contain EG and DEG in molar ratios of 0.25/1.75, 0.5/1.5, and 0.75/1.25 (feed composition), respectively. Cured formulations exhibit characteristics between the original samples without synergistic effects (Table 3 and Fig. S8† in the ESI).

## Conclusions

This study outlines a sustainable method for the synthesis of UP resin using post-consumer PET and bio-based raw materials. The process involves glycolysis of PET with DEG, followed by condensation polymerization with IA and dilution with RD. The mechanical properties of the cured resin were adjusted by varying the EG/DEG molar ratio during the glycolysis step. When the EG/DEG ratio was 1/1, no side products were formed, except condensation water. Lower molar ratios led to the release of EG during glycolysis, but it could be easily recovered by distillation for reuse. The introduction of IA into the polyester structure, instead of more common fumarates, allowed the use of RD based on methacrylate and itaconate in the resin formulation. Cured solutions in MMA and DMI exhibited promising mechanical properties comparable to those of relevant STY formulations. Our preference for DMI is highlighted here not only for sustainability reasons but also because of its considerably lower volatility and flammability.

In conclusion, the technology presented here serves as an example of upcycling PET waste, not only addressing the problem of plastic waste, but also offering a solution to replace hazardous chemicals in the market of UP resins.

## Experimental section

### Materials

Butyl methacrylate (BMA), hydroquinone, itaconic acid (IA), methanol, methyl methacrylate (stab. with topanol; MMA), styrene (stabilized; STY), and toluene-4-sulfonic acid monohydrate (PTSA) were supplied from Thermo Fisher Scientific. Diethylene glycol (DEG), sulfuric acid (96%), sodium sulfate (anhydrous) and tetrahydrofuran (THF) were supplied from Penta. Ethyl acetate, butanol, and toluene were supplied from Lach-Ner. Dibutyltin oxide (DBTO) was obtained from PMC Group. Butanone peroxide (32% in a mixture of dimethyl phthalate and dicetone alcohol; MEKP) was obtained from Stachema. Oxidovanadium(iv) dibutylphosphate (10.6% V) was synthesized as described elsewhere.^45^ Colorless PET bottles without cups and labels were washed with distilled water, cut to 2 × 2 cm^2^ flakes, and dried in an oven at 50 °C for 1 h and characterized by measurements of intrinsic viscosity [*η*]. The value of 0.77 dL g^−1^ was determined using an Ubbelohde viscometer in a 3/2 mixture (w/w) of phenol/1,1,2,2-tetrachloroethane at 30 °C.

### Syntheses

#### Synthesis of dimethyl itaconate (DMI)

A mixture of itaconic acid (200 g, 1.54 mol) in methanol (700 mL) was treated with hydroquinone (0.1 g) and sulfuric acid (50 mL). The reaction mixture was heated under reflux for 4.5 h. The resulting mixture was cooled and kept at −20 °C overnight while the crude product precipitated. The precipitate was filtered off and washed with cold water until the filtrate was pH-neutral. The crude DMI was dissolved in ethyl acetate (∼250 mL), washed with brine (2 × 100 mL), and dried with anhydrous sodium sulfate. The volatiles were evaporated under vacuum and the final product was stored in a fridge, where it slowly crystallized. Yield: 170 g (1.08 mol, 70%). Colorless solid. The analytical and spectroscopic data were consistent with those published elsewhere.^46^

#### Synthesis of dibutyl itaconate (DBI)

The reaction was carried out as described for DMI but with itaconic acid (100 g, 0.77 mol), butanol (114 g, 1.54 mol), hydroquinone (0.05 g) and sulfuric acid (25 mL). The crude product was washed with cold water in a separatory funnel until the washing water was neutral. The product was dried with anhydrous sodium sulfate and stored in a refrigerator. Yield: 183 g (0.75 mol, 97%). Analytical and spectroscopic data were consistent with those published elsewhere.^46^

#### Synthesis of GL1

A mixture of PET flakes (400 g, 2.08 mol of the repeat units), DEG (221 g, 2.08 mol) and DBTO (0.6 g) was heated to 245 °C and vigorously stirred with an anchor stirrer (300 rpm) for 4 h. The product was characterized by ^1^H NMR spectroscopy and SEC chromatography and was used without further purification.

#### Synthesis of GL2

A mixture of PET flakes (400 g, 2.08 mol of the repeat units), DEG (332 g, 3.13 mol) and DBTO (0.7 g) was heated to 245 °C and vigorously stirred with an anchor stirrer (300 rpm). The EG produced was continuously distilled from the reaction mixture. Glycolysis was stopped when 71.9 mL (1.04 mol) of EG was separated. The product was characterized by ^1^H NMR spectroscopy and SEC chromatography and was used without further purification.

#### Synthesis of GL3

The reaction was carried out as described for GL2, but with PET flakes (400 g, 2.08 mol of the repeat units), DEG (442 g, 4.16 mol) and DBTO (0.8 g). Glycolysis was stopped when 143.9 mL (2.08 mol) of EG was separated. The product was characterized by ^1^H NMR spectroscopy and SEC chromatography and was used without further purification.

#### Synthesis of UPET1

A mixture of GL1 (150 g, 0.503 mol of TPA units, 1.01 mol of diol units), itaconic acid (65.5 g, 0.503 mol), DBTO (0.2 g), PTSA (0.5 g) and hydroquinone (0.05 g) was heated under nitrogen inlet while stirred with anchor stirrer (300 rpm). At 160 °C, the homogeneous reaction mixture was treated with toluene (10 mL) and the nitrogen flow was stopped. The water produced was separated by the Dean–Stark apparatus. When the acid value fell below 50 mg g^−1^ of KOH, the mixture temperature was kept at 160 °C and the volatiles were evaporated under vacuum. After collation at 80 °C, the sample was collected for analyzes and the polyester was diluted by the given RD. We note that DMI was melted in a water bath before use.

#### Synthesis of UPET2

The reaction was carried out as described for GL1, but with GL2 (150 g, 0.469 mol of TPA units, 0.938 mol of diol units), itaconic acid (61.0 g, 0.469 mol), DBTO (0.2 g), PTSA (0.5 g) and hydroquinone (0.05 g).

#### Synthesis of UPET3

The reaction was carried out as described for GL1 but with GL3 (150 g, 0.439 mol of TPA units, 0.878 mol of diol units), itaconic acid (57.1 g, 0.439 mol), DBTO (0.2 g), PTSA (0.5 g) and hydroquinone (0.05 g).

### Methods

#### Curing and preparation of test specimens

The polyester resin (60 g) was treated with oxidovanadium(iv) dibutylphosphate (56 mg) and thoroughly stirred. The mixture was treated with MEKP (250 μL) and stirred vigorously to obtain a homogenous mixture. After degassing in a centrifuge (4000 rpm for 2 min), the resin was then poured into silicone molds cured at room temperature overnight. The next day, the specimens were post-cured in an oven under the following conditions: at 50 °C for 1 h, 70 °C for 1 h, 90 °C for 1 h. Finally, all samples were slowly cooled to ambient temperature to avoid thermal stress. Three types of specimens were used in this study. Specimen A: rectangular cuboid of dimensions 50 × 6 × 3 mm^3^; Specimen B: dogbone-shaped specimen of 2 mm thickness, 47.5 mm total length, 14.7 mm wide gripping area, and 12.5 × 5 mm^2^ thinned middle section dimensions; Specimen C, cylinder of high 20 mm and diameter 12 mm. Detailed schematic drawings of test specimens are given in Fig. S9 in the ESI.†

#### Gel content

The specimens A were ground cryogenically in an impact mill IKA A10 Basic cooled with liquid nitrogen. The ground material (∼1 g) was precisely weighted (*m*_1_), treated with THF (30 mL) and stirred in a closed Erlenmeyer flask overnight. The solids were filtered, dried in an oven at 50 °C overnight, and weighted (*m*_2_). The gel content (in%) was calculated according to: GEL = (*m*_2_/*m*_1_) × 100.

#### NMR spectroscopy

The ^1^H NMR and ^1^H–^1^H COSY NMR spectra of the samples, dissolved in CDCl_3_, were collected on Bruker Avance 400 and Bruker Avance 500 spectrometers at room temperature. Chemical shifts are reported in ppm relative to tetramethylsilane.

#### Chromatography

Size exclusion chromatography (SEC) was performed on a Waters liquid chromatograph Alliance e2695 with a refractive index detector 2414 and two Agilent Mixed-E columns 300 × 7.5 mm^2^ (GL1–GL3) or two Agilent Mixed-C columns 300 × 7.5 mm^2^ (UPET1–UPET3) THF was used as mobile phase at a flow rate of 1 mL min^−1^. The samples were dissolved in THF (*c* = 3 mg mL^−1^) and filtered through 0.45 μm filters (injected volume = 100 μL). The calibration on polystyrene standards covered the molar mass ranges of 162 to 30 000 g mol^−1^ (Mixed-E columns) and 162 to 6 × 10^6^ g mol^−1^ (Mixed-C columns).

#### Viscosity

It was measured on a Höppler's Rheo-Viscometer (Veb Prufgerate-Werk) with cells 0.1 (*k* = 0.10312) and 1 (*k* = 1.05758) and 200 g of weight at a temperature of 25 °C.

#### Thermomechanical properties

Dynamic mechanical analysis was performed on specimens A using a DX045 device (RMI) in a single fixed point cantilever configuration with a span between clamps of 11 mm, a deviation of ±0.15 mm, and a frequency of 1 Hz. Measurements were made in the range −60 °C to 150 °C and a heating rate of 3 °C min^−1^. The temperature of the glass transition (*T*_g_) was determined as the tan *δ* peak maximum. The crosslink density (*ν*_e_) was calculated using the equation: *ν*_e_ = *G*′/*RT*, where *G*′ is the storage modulus of the networks in the rubbery plateau (at *T* = 145 °C), *R* is the universal gas constant, and *T* is the absolute temperature in Kelvin.

#### Mechanical tests

Tensile testing was performed on specimens B using a universal testing machine Instron 1122 (Bluehill Universal) with a head span of 30 mm at a head speed of 5 mm min^−1^.

Flexural testing was carried out on specimens A using a universal testing machine Autograph AGS-X 50 kN (Shimadzu) in a three-point bending configuration with a support span of 40 mm at a head speed of 1 mm min^−1^. The experiment was stopped when the specimen ruptured or the force exerted on the specimen was less than 10 N.

Compressive testing was conducted in specimens C using a universal testing machine Autograph AGS-X 50 kN (Shimadzu) at a head speed of 1 mm min^−1^.

Impact toughness was measured according to Charpy on a Plastic Pendulum Impact Tester 501 J-3 (Shenzhen Wance Testing Machine) with a support span of 40 mm. Testing was carried out on unnotched specimens A.

#### Microscopy

Scanning electron microscopy (SEM) was performed on fractured specimens analyzed after impact toughness measurements using a scanning electron microscope JSM 5500-LV (Jeol) under high vacuum, acceleration voltage of 10 kV, work distance 12–13 mm, spot size of 20. Secondary electrons emitted were detected. The fracture surfaces were metalized by atomic deposition of gold.

## Data availability

Data for this paper are available for download from http://dx.doi.org/10.5281/zenodo.10155550.

## Author contributions

David Rubeš contributed to the conceptualization of the project and performed all reaction experiments, characterization by DMA, and mechanical tests. He contributed to the writing of the original manuscript. Jaromír Vinklárek supervised the project. Štěpán Podzimek characterized polyesters by SEC. Jan Honzíček conceptualized the project, interpreted NMR data, wrote the original manuscript, and edited the manuscript.

## Conflicts of interest

There are no conflicts to declare.

## Supplementary Material

RA-014-D3RA08500G-s001

## References

[cit1] Millican J. M., Agarwal S. (2021). Plastic Pollution: A Material Problem?. Macromolecules.

[cit2] Vogt B. D., Stokes K. K., Kumar S. K. (2021). Why is Recycling of Postconsumer Plastics so Challenging?. ACS Appl. Polym. Mater..

[cit3] Chen Y., Awasthi A. K., Wei F., Tan Q., Li J. (2021). Single-use plastics: Production, usage, disposal, and adverse impact. Sci. Total Environ..

[cit4] Nait-Ali L. K., Colin X., Bergeret A. (2011). Kinetic analysis and modelling of PET macromolecular changes during its mechanical recycling by extrusion. Polym. Degrad. Stab..

[cit5] Kalmykova Y., Sadagopan M., Rosado L. (2018). Circular economy – From review of theories and practices to development of implementation tools. Resour., Conserv. Recycl..

[cit6] Payne J., Jones M. D. (2021). The Chemical Recycling of Polyesters for a Circular Plastics Economy: Challenges and Emerging Opportunities. ChemSusChem.

[cit7] Tournier V., Topham C. M., Gilles A., David B., Folgoas C., Moya-Leclair E., Kamionka E., Desrousseaux M.-L., Texier H., Gavalda S., Cot M., Guémard E., Dalibey M., Nomme J., Cioci G., Barbe S., Chateau M., André I., Marty S. D. A. (2020). An engineered PET depolymerase to break down and recycle plastic bottles. Nature.

[cit8] Cao Z., Fu X., Li H., Pandit S., Amombo Noa F. M., Öhrström L., Zelezniak A., Mijakovic I. (2023). Synthesis of Metal-Organic Frameworks through Enzymatically Recycled Polyethylene Terephthalate. ACS Sustainable Chem. Eng..

[cit9] Barnard E., Arias J. J. R., Thielemans W. (2021). Chemolytic depolymerisation of PET: a review. Green Chem..

[cit10] Ranganathan P., Chen Y.-H., Rwei S.-P., Lee Y.-H. (2022). Biomass upcycling of waste rPET to higher-value new-easy-recyclable microcellular thermoplastic (co)polyamide foams and hot-melt adhesives. Mater. Today Adv..

[cit11] Duque-Ingunza I., López-Fonseca R., de Rivas B., Gutiérrez-Ortiz J. I. (2013). Synthesis of unsaturated polyester resin from glycolyzed postconsumer PET wastes. J. Mater. Cycles Waste Manage..

[cit12] Pu M., Zhou X., Liu X., Fang C., Wang D. (2023). A facile, alternative and sustainable feedstock for transparent polyurethane elastomers from chemical recycling waste PET in high-efficient way. Waste Manage..

[cit13] Ma Y., Lei R., Yang X., Yang F. (2020). Eco-friendly Waterborne Alkyd Resin from Polyethylene Terephthalate Waste. J. Polym. Environ..

[cit14] Ghosal K., Nayak C. (2022). Recent advances in chemical recycling of polyethylene terephthalate waste into value added products for sustainable coating solutions – hope vs. hype. Mater. Adv..

[cit15] Kosloski-Oh S. C., Wood Z. A., Manjarrez Y., Pablo de los Rios J., Fieser M. E. (2021). Catalytic methods for chemical recycling or upcycling of commercial polymers. Mater. Horiz..

[cit16] Jehanno C., Alty J. W., Roosen M., De Meester S., Dove A. P., Chen E. Y.-X., Leibfarth F. A., Sardon H. (2022). Critical advances and future opportunities in upcycling commodity polymers. Nature.

[cit17] Hou Q., Zhen M., Qian H., Nie Y., Bai X., Xia T., Rehman M. L. U., Li Q., Ju M. (2021). Upcycling and catalytic degradation of plastic wastes. Cell Rep. Phys. Sci..

[cit18] Papadopoulos L., Malitowski N. M., Bikiaris D., Robert T. (2023). Bio-based additive manufacturing materials: An in-depth structure-property relationship study of UV-curing polyesters from itaconic acid. Eur. Polym. J..

[cit19] Afewerki S., Edlund U. (2023). Engineering an All-Biobased Solvent- and Styrene-Free Curable Resin. ACS Polym. Au.

[cit20] Panic V. V., Seslija S. I., Popovic I. G., Spasojevic V. D., Popovic A. R., Nikolic V. B., Spasojevic P. M. (2017). Simple One-Pot Synthesis of Fully Biobased Unsaturated Polyester Resins Based on Itaconic Acid. Biomacromolecules.

[cit21] Dai Z. H., Yang Z. W., Chen Z. W., Zhao Z. X., Lou Y. J., Zhang Y. Y., Liu T. X., Fu F. Y., Fu Y. Q., Liu X. D. (2018). Fully Biobased Composites of an Itaconic Acid Derived Unsaturated Polyester Reinforced with Cotton Fabrics. ACS Sustainable Chem. Eng..

[cit22] Fidanovski B. Z., Spasojevic P. M., Panic V. V., Seslija S. I., Spasojevic J. P., Popovic J. G. (2018). Synthesis and characterization of fully bio-based unsaturated polyester resins. J. Mater. Sci..

[cit23] Rorrer N. A., Dorgan J. R., Vardon D. R., Martinez C. R., Yang Y., Beckham G. T. (2016). Renewable Unsaturated Polyesters from Muconic Acid. ACS Sustainable Chem. Eng..

[cit24] Rorrer N. A., Vardon D. R., Dorgan J. R., Gjersing E. J., Beckham G. T. (2017). Biomass-derived monomers for performancedifferentiated fiber reinforced polymer composites. Green Chem..

[cit25] Rorrer N. A., Nicholson S., Carpenter A., Biddy M. J., Grundl N. J., Beckham G. T. (2019). Combining Reclaimed PET with Bio-based Monomers Enables Plastics Upcycling. Joule.

[cit26] Hofmann M. A., Shahid A. T., Garrido M., Ferreira M. J., Correia J. R., Bordado J. C. (2022). Biobased Thermosetting Polyester Resin for High-Performance Applications. ACS Sustainable Chem. Eng..

[cit27] Dai J., Ma S., Teng N., Dai X., Shen X., Wang S., Liu X., Zhu J. (2017). 2,5-Furandicarboxylic Acid- and Itaconic Acid-Derived Fully Biobased Unsaturated Polyesters and Their Cross-Linked Networks. Ind. Eng. Chem. Res..

[cit28] Krämer H. , Polyester Resins, Unsaturated, in Ullmann's Encyclopedia of Industrial Chemistry, Wiley-VCH Verlag, Weinheim, 2000, p. 14356007, 10.1002/14356007.a21_217

[cit29] Rubeš D., Vinklárek J., Prokůpek L., Podzimek Š., Honzíček J. (2023). Styrene-free unsaturated polyester resins derived from itaconic acid curable by cobalt-free accelerators. J. Mater. Sci..

[cit30] Huff J., Infante P. F. (2011). Styrene exposure and risk of cancer. Mutagenesis.

[cit31] Cushman J. R., Rausina G. A., Cruzan G., Gilbert J., Williams E., Harrass M. C., Sousa J. V., Putt A. E., Garvey N. A., Laurent J. P. St., Hoberg J. R., Machado M. W. (1997). Ecotoxicity Hazard Assessment of Styrene. Ecotoxicol. Environ. Saf..

[cit32] Zhao L., Zhu W., Papadaki M. I., Mannan M. S., Akbulut M. (2019). Probing into Styrene Polymerization Runaway Hazards: Effects of the Monomer Mass Fraction. ACS Omega.

[cit33] Lima M. S., Costa C. S. M. F., Coelho J. F. J., Fonseca A. C., Serra A. C. (2018). A simple strategy toward the substitution of styrene by sobrerol-based monomers in unsaturated polyester resins. Green Chem..

[cit34] Arnaud S. P., Malitowski N. M., Casamayor K. M., Robert T. (2021). Itaconic Acid-Based Reactive Diluents for Renewable and Acrylate-Free UV-Curing Additive Manufacturing Materials. ACS Sustainable Chem. Eng..

[cit35] Yu A. Z., Serum E. M., Renner A. C., Sahouani J. M., Sibi M. P., Webster D. C. (2018). Renewable Reactive Diluents as Practical Styrene Replacements in Biobased Vinyl Ester Thermosets. ACS Sustainable Chem. Eng..

[cit36] Dai Z. H., Li Q., Chen Z. W., Shawon R. K., Zhu Y. Y., Lv H. F., Fu F. Y., Zhu Y. F., Fu Y. Q., Liu X. D. (2020). Reactive Diluent Derived from Ferulic Acid for the Preparation of a Fully Biobased Unsaturated Polyester Resin. ACS Sustainable Chem. Eng..

[cit37] Robert T., Friebel S. (2016). Itaconic acid – a versatile building block for renewable polyesters with enhanced functionality. Green Chem..

[cit38] Varriale L., Ulber R. (2023). Fungal-Based Biorefinery: From Renewable Resources to Organic Acids. ChemBioEng Rev..

[cit39] Kadkin O., Osajda K., Kaszynski P., Barber T. A. (2003). Polyester polyols: Synthesis and characterization of diethylene glycol terephthalate oligomers. J. Polym. Sci., Part A: Polym. Chem..

[cit40] Farmer T. J., Castle R. L., Clark J. H., Macquarrie D. J. (2015). Synthesis of Unsaturated Polyester Resins from Various Bio-Derived Platform Molecules. Int. J. Mol. Sci..

[cit41] European Chemicals Agency , https://www.echa.europa.eu/, accessed November 2023

[cit42] Sollka L., Lienkamp K. (2021). Progress in the Free and Controlled Radical Homo- and Co-Polymerization of Itaconic Acid Derivatives: Toward Functional Polymers with Controlled Molar Mass Distribution and Architecture. Macromol. Rapid Commun..

[cit43] Claudy P., Létoffé J. M., Camberlain Y., Pascault J. P. (1983). Glass Transition of Polystyrene Versus Molecular Weight. Polym. Bull..

[cit44] Fernández-Garcia M., Madruga E. L. (1997). Glass transitions in dimethyl and di-n-butyl poly(itaconate ester)s and their copolymers with methyl methacrylate. Polymer.

[cit45] Link G. , Edelmann D. and Stumpp E., Use of special vanadium compounds as siccatives for oxidatively drying lacquers, US Pat.Patent US 6063841A, May 16, 2000

[cit46] Boschert D., Schneider-Chaabane A., Himmelsbach A., Eickenscheidt A., Lienkamp K. (2018). Synthesis and Bioactivity of Polymer-Based Synthetic Mimics of Antimicrobial Peptides (SMAMPs) Made from Asymmetrically Disubstituted Itaconates. Chem. - Eur. J..

