# Supplementary material for: Bio-based unsaturated polyester resin from post-consumer PET
Source: RSC Adv. 2024 Mar 13;14(12):8536–47. doi: 10.1039/d3ra08500g (PMC10934379; doi:10.1039/d3ra08500g)
Supplement: RA-014-D3RA08500G-s001 [file RA-014-D3RA08500G-s001.pdf]

Electronic Supplementary Material (ESI) for RSC Advances.
This journal is © The Royal Society of Chemistry 2024

Supporting Information for:

# Bio-based Unsaturated Polyester Resin from Post-Consumer PET.

David Rubeš,[a] Jaromír Vinklárek,[b] Štěpán Podzimek,[a] and Jan Honzíček[b,*]

[a] Institute of Chemistry and Technology of Macromolecular Materials, Faculty of Chemical Technology, University of Pardubice, Studentská 573, 532 10 Pardubice, Czech Republic

[b] Department of General and Inorganic Chemistry, Faculty of Chemical Technology, University of Pardubice, Studentská 573, 532 10 Pardubice, Czech Republic

P.2 **Figure S1.** SEC chromatogram of GL1.

P.2 **Figure S2.** SEC chromatogram of GL2.

P.2 **Figure S3.** SEC chromatogram of GL3.

P.3 **Figure S4.** SEC chromatogram of UPET1

P.3 **Figure S5.** SEC chromatogram of UPET2

P.4 **Figure S6.** SEC chromatogram of UPET3

P.4 **Table S1.** Composition of PET glycolysis products.

P.4 **Table S2.** Composition of synthesized UPs.

P.5 **Figure S7.** Tensile properties of cured polyester formulations.

P.5 **Figure S8.** Properties of UPET1/3-DMI40 blends.

P.6 **Figure S9.** Testing specimen dimensions

P.6 **Table S3.** Biobased and recycled contents

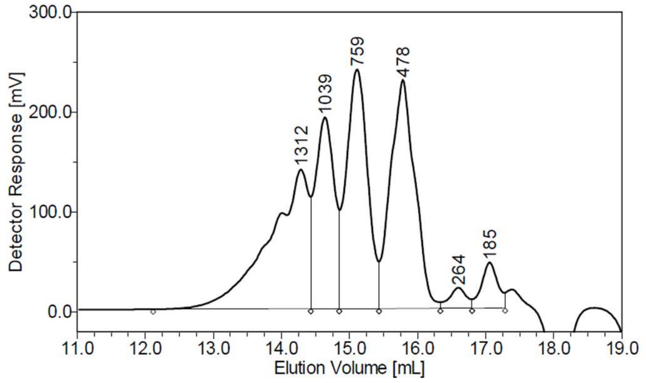


| | MP (Daltons) | Area | % Area | Height | Retention Time (min) |
|---|---|---|---|---|---|
| 1 | 1312 | 5610094 | 26.2 | 139409 | 14.286 |
| 2 | 1039 | 3715384 | 17.3 | 191476 | 14.639 |
| 3 | 759 | 5250889 | 24.5 | 239247 | 15.111 |
| 4 | 478 | 5739812 | 26.8 | 228687 | 15.783 |
| 5 | 264 | 360054 | 1.7 | 20673 | 16.599 |
| 6 | 185 | 757020 | 3.5 | 45675 | 17.057 |

**Figure S1.** SEC chromatogram of GL1.

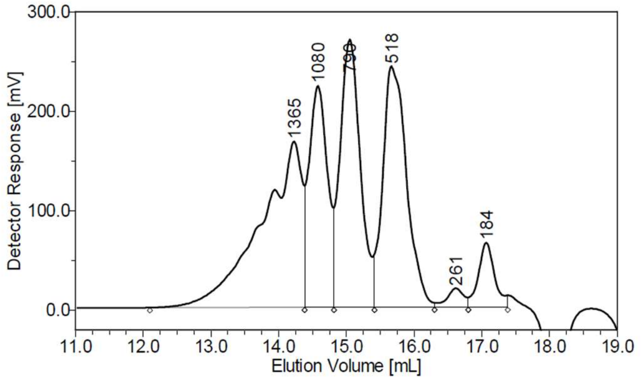


| | MP (Daltons) | Area | % Area | Height | Retention Time (min) |
|---|---|---|---|---|---|
| 1 | 1365 | 7023270 | 28.9 | 166813 | 14.227 |
| 2 | 1080 | 4268843 | 17.6 | 222684 | 14.580 |
| 3 | 790 | 5651402 | 23.3 | 269390 | 15.050 |
| 4 | 518 | 5911522 | 24.4 | 242389 | 15.667 |
| 5 | 261 | 330711 | 1.4 | 19174 | 16.615 |
| 6 | 184 | 1076396 | 4.4 | 64821 | 17.065 |

**Figure S2.** SEC chromatogram of GL2.

GPC Results

| | Mn | Mw | Mz | Polydispersity | Area (µV*sec) | End Time (min) |
|---|---|---|---|---|---|---|
| 1 | 1349 | 2333 | 3542 | 1.73 | 2.37e+007 | 16.95 |

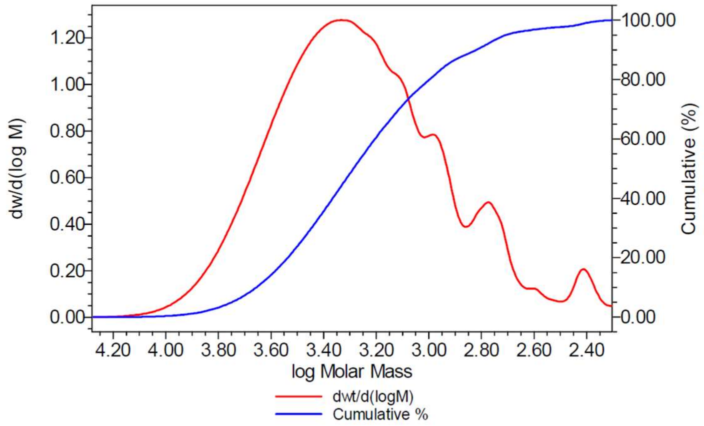


**Figure S3.** SEC chromatogram of GL3.

GPC Results

| | Mn | Mw | MP | Mz | Mz+1 | Polydispersity | Area (µV*sec) | End Time (min) |
|---|---|---|---|---|---|---|---|---|
| 1 | 2153 | 4762 | 3866 | 8668 | 13727 | 2.21 | 2.42e+007 | 18.28 |

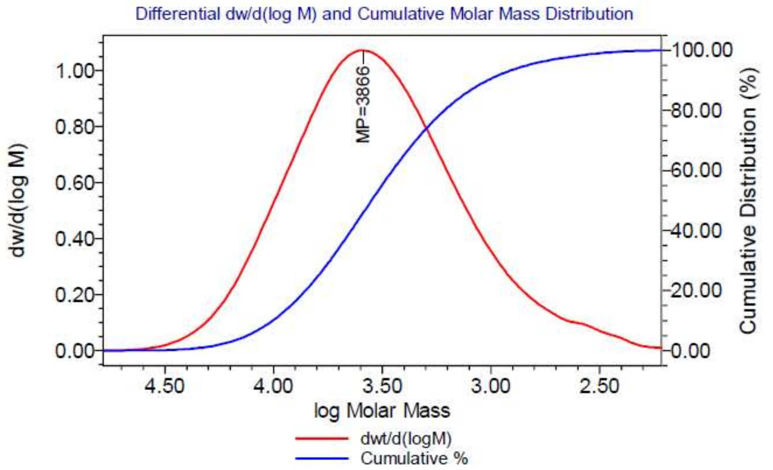


**Figure S4.** SEC chromatogram of UPET1.

GPC Results

| | Mn | Mw | MP | Mz | Mz+1 | Polydispersity | Area (µV*sec) | End Time (min) |
|---|---|---|---|---|---|---|---|---|
| 1 | 2134 | 4332 | 3541 | 8021 | 14435 | 2.03 | 2.25e+007 | 18.07 |

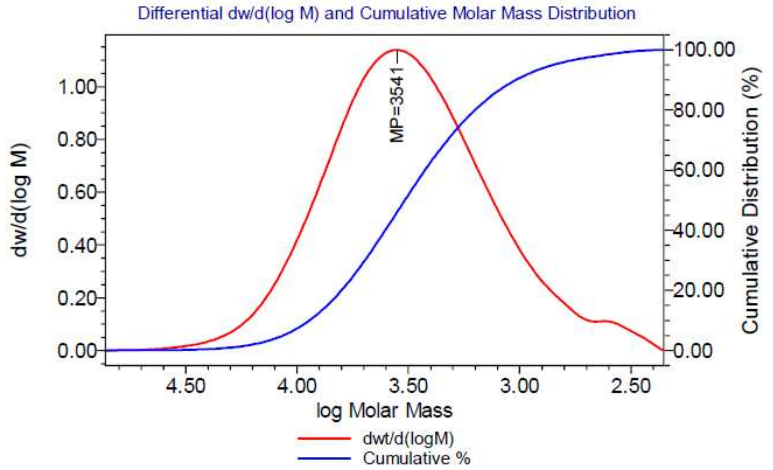


**Figure S5.** SEC chromatogram of UPET2.

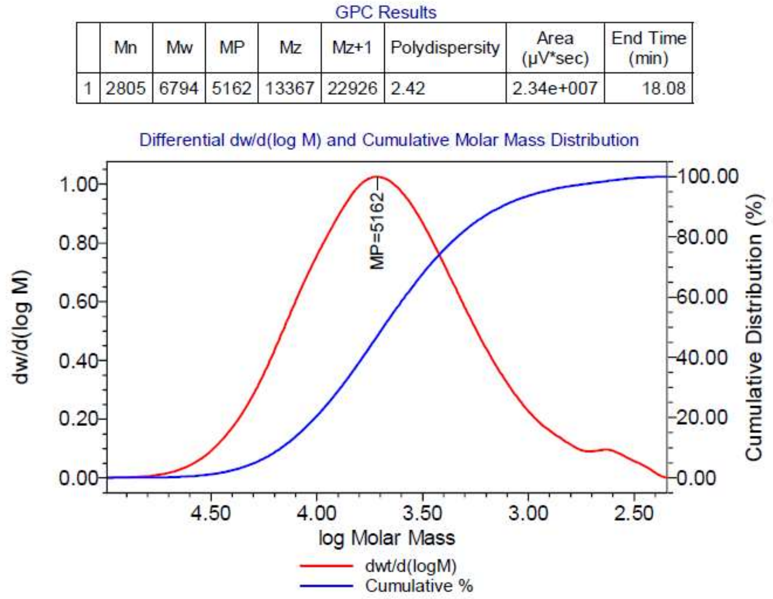


**Figure S6.** SEC chromatogram of UPET3.

**Table S1.** Composition of PET glycolysis products.

| Glycolysate | Feed composition [a] | Theoretical segment distribution [b,c] | Experimental segment distribution [b,d] |
|---|---|---|---|
| GL1 | 1/1/1 | 1/1/2/2/1/1 | 0.77/1.12/2.13/2.08/0.95/0.94 |
| GL2 | 1/0.5/1.5 | 0.33/1/0.66/2/0.33/1 | 0.33/0.92/0.85/1.88/0.44/0.90 |
| GL3 | 1/0/2 | 0/1/0/2/0/1 | 0.06/0.67/0.13/2.73/0.00/0.42 |

[a] Molar ratio of the building blocks TPA/EG/DEG. [b] Molar ratio of TPA-EG-TPA/TPA-DEG-TPA/TPA-EG-OH/TPA-DEG-OH/EG/DEG segments given in Scheme 1. [c] Based on the statistical distribution of equally reactive hydroxyl groups. [d] Determined by $^1$H NMR spectroscopy.

**Table S2.** Composition of synthesized UPs.

| Polyester | Feed composition (molar ratio) [a] | Polyester composition (molar ratio) [b] |
|---|---|---|
| UPET1 | 1/1/1/1 | 0.99/1.00/0.80/1.24/0.04/0.06 |
| UPET2 | 1/1/0.5/1.5 | 0.99/1.00/0.42/1.57/0.04/0.05 |
| UPET3 | 1/1/0/2 | 0.71/1.00/0.14/1.45[c]/0.04/0.05 |

[a] Feed composition IA/TPA/EG/DEG. [b] Molar ratio of the IA/TPA/EG/DEG/MES/OA segments determined by $^1$H NMR spectroscopy. [c] Covers higher oligomers of EG.

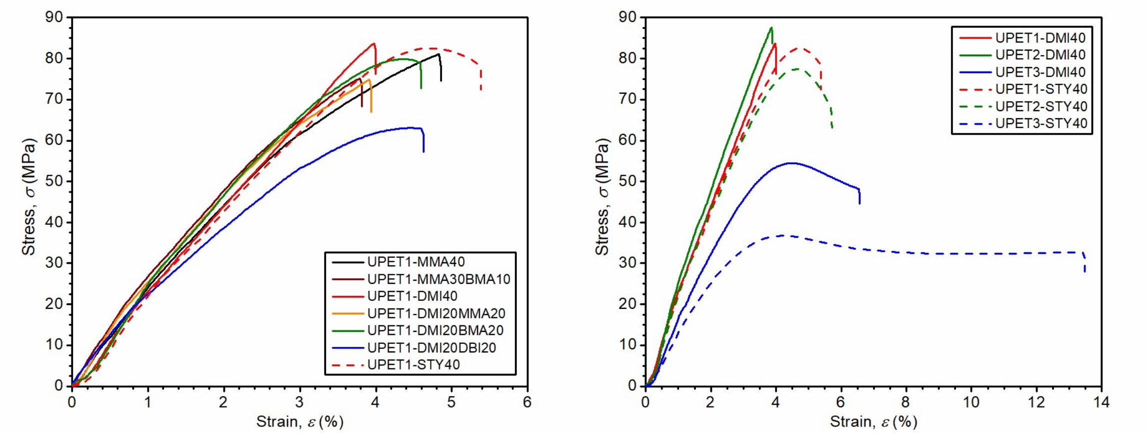


**Figure S7.** Tensile properties of cured polyester formulations.

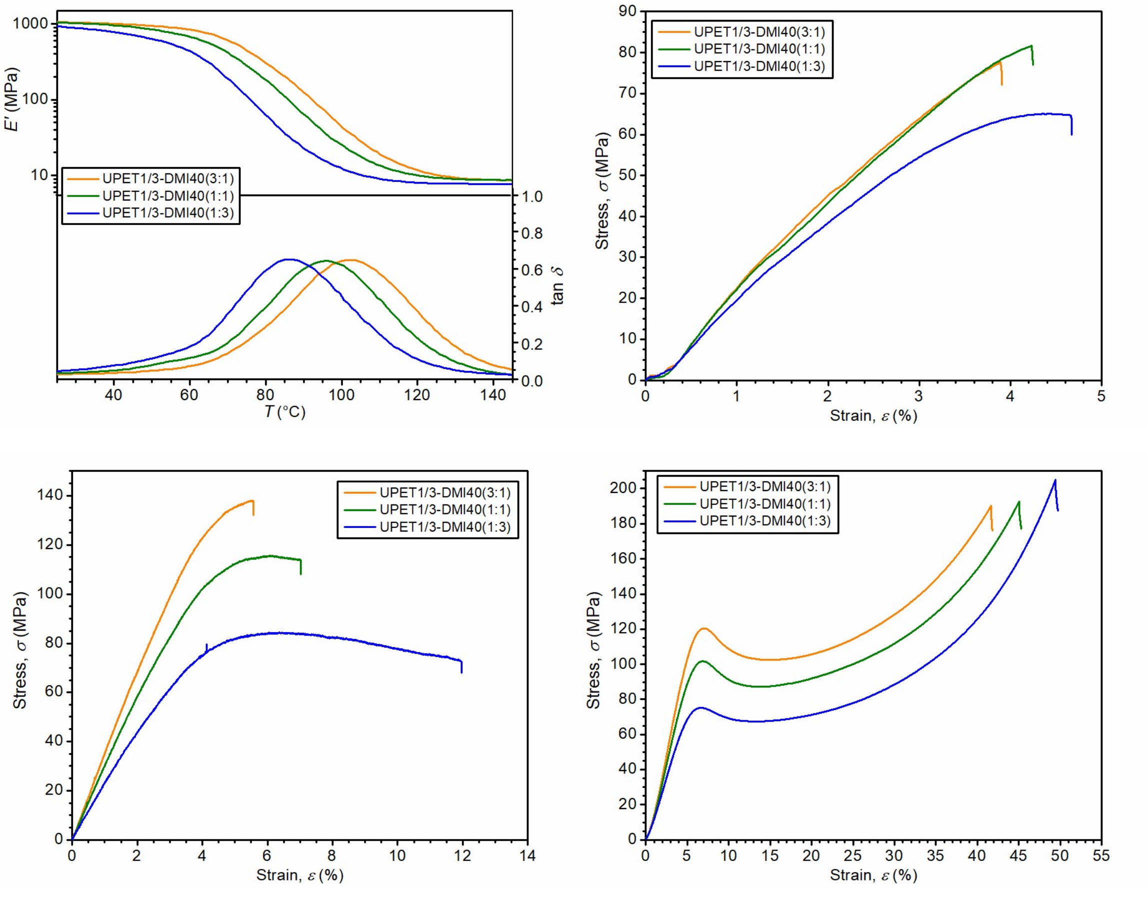


**Figure S8**. Properties of UPET1/3-DMI40 blends: DMA analysis (top left), tensile (top right), flexure (bottom left) and compression measurements (bottom right).

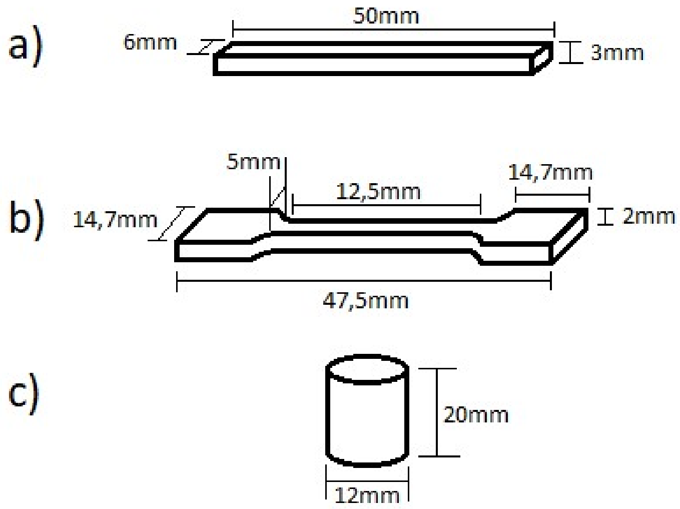


**Table S3.** Bio-based and recycled contents in UP prepolymer and formulations of UP resins.[a]

| | UP | | UP resin | |
|---|---|---|---|---|
| | Biobased | Recycled | Biobased | Recycled |
| UPET1-MMA40 | 51.0 | 49.0 | 43.0 | 29.4 |
| UPET1-MMA30BMA10 | 51.0 | 49.0 | 45.1 | 29.4 |
| UPET1-DMI40 | 51.0 | 49.0 | 70.6 | 29.4 |
| UPET1-DMI20MMA20 | 51.0 | 49.0 | 56.8 | 29.4 |
| UPET1-DMI20BMA20 | 51.0 | 49.0 | 60.9 | 29.4 |
| UPET1-DMI20DBI20 | 51.0 | 49.0 | 70.6 | 29.4 |
| UPET1-STY40 | 51.0 | 49.0 | 30.6 | 29.4 |
| UPET2-DMI40 | 60.9 | 39.1 | 76.5 | 23.5 |
| UPET2-STY40 | 60.9 | 39.1 | 36.5 | 23.5 |
| UPET3-DMI40 | 69.7 | 30.3 | 81.8 | 18.2 |
| UPET3-STY40 | 69.7 | 30.3 | 41.8 | 18.2 |
| UPET1/3-DMI40(3:1) | 55.7 | 44.3 | 73.4 | 26.6 |
| UPET1/3-DMI40(1:1) | 60.4 | 39.6 | 76.2 | 23.8 |
| UPET1/3-DMI40(1:3) | 65.1 | 34.9 | 79.0 | 21.0 |

[a] Given in wt.%.
